# Trajectories of maternal depressive symptoms and offspring’s risk behavior in early adolescence: data from the 2004 Pelotas birth cohort study

**DOI:** 10.1186/s12888-020-03026-9

**Published:** 2021-01-07

**Authors:** Ana Beatriz Bozzini, Jessica Mayumi Maruyama, Tiago N. Munhoz, Aluísio J. D. Barros, Fernando C. Barros, Iná S. Santos, Alicia Matijasevich

**Affiliations:** 1 Departamento de Medicina Preventiva, Faculdade de Medicina FMUSP, Universidade de São Paulo, São Paulo, Brazil; 2 Postgraduate Program in Epidemiology, Federal University of Pelotas, Pelotas, Brazil; 3 Faculty of Psychology, Federal University of Pelotas, Pelotas, Brazil; 4 Post-graduate Program in Health and Behavior, Catholic University of Pelotas, Pelotas, Brazil; 5 Postgraduate Program in Pediatrics and Child Health, School of Medicine, Pontifical Catholic University of Rio Grande do Sul, Porto Alegre, Brazil

**Keywords:** Adolescent behavior, Risk -taking, Cohort studies

## Abstract

**Background:**

This longitudinal study explored the relationship between trajectories of maternal depressive symptoms and offspring’s risk behavior in adolescence contributing to an extremely scarce literature about the impacts of maternal depression trajectories on offspring risk behaviors.

**Methods:**

We included 3437 11-year-old adolescents from the 2004 Pelotas Birth Cohort Study. Trajectories of maternal depressive symptoms were constructed using Edinburgh Postnatal Depression Scale (EDPS) from age 3 months to 11 years. We identified five trajectories of maternal depressive symptoms: “low” “moderate low”, “increasing”, “decreasing”, and “chronic high”. The following adolescent outcomes were identified via self-report questionnaire and analyzed as binary outcome –yes/no: involvement in fights and alcohol use at age 11. We used logistic regression models to examine the effects of trajectories of maternal depressive symptoms on offspring’s risk behavior adjusting for potential confounding variable.

**Results:**

Alcohol use and/or abuse as well as involvement in fights during adolescence, were not significantly associated with any specific trajectory of maternal depressive symptoms neither in the crude nor in the adjusted analyses.

**Conclusion:**

Alcohol use and involvement in fights at age 11 were not associated with any specific trajectory of maternal depression.

**Supplementary Information:**

The online version contains supplementary material available at 10.1186/s12888-020-03026-9.

## Background

Adolescence is marked by complex biological and psychological transformations that potentially could lead to risky, health-compromising behaviors and cause physical and mental impairments. These behaviors include exposure to violence, sexual risk behavior and the use of tobacco, alcohol and illicit substances [1]. Many of these activities are often sporadic, but if a consolidating pattern of risky behavior is not early identified and effectively monitored, the individual’s health as well as her/his social and family ties may be severely harmed. There is an increased interest in studying risk behavior in adolescents due to its correlation with morbidity and mortality in adult life [1, 2].

The majority of studies about risk behavior in adolescence report current associated factors, particularly sociodemographic determinants such as neighborhood characteristics, peer influence and family’s socioeconomic status [3, 4]. Early determinants of risk behaviors are poorly explored, although it is well established that early infancy experiences can leave a lasting signature affecting emerging brain architecture and long-term health [5]. The literature describes three sets of early determinants that impact a child’s future behavior: prenatal exposures such as maternal depression, tobacco, alcohol, caffeine and others substances’ use during pregnancy; birth conditions such as prematurity and low birth weight; and adverse experiences during early life, such as maltreatment, neglect, family violence, adoption, and maternal or caregiver’s emotional problems - including maternal postpartum depression [6–9].

Maternal depression after childbirth is a serious public health problem and has been explored in longitudinal studies as a predictor of emotional and mental problems in offspring [10–12]. Evidence suggests that children born from untreated depressed mothers (compared to mothers without maternal depression) are at greater risk to present behavioral inhibition, emotional maladjustment, violent behavior, externalizing problems, and psychiatric disorders in adolescence [13, 14]. Recently, studies evaluated not only ‘severity’ or ‘timing’, but also the ‘course’ of maternal depression and its impact on offspring’s emotional and behavioral outcomes [11, 15, 16].

Studies about trajectories of maternal depression, in general, identify two to six trajectories [11, 13, 15, 16], from none or minimal depressive symptoms to high chronic symptomatology. They mostly reported that children from mothers belonging to chronic and severe depressive symptoms trajectories were at more risk to present emotional problems when compared to those from mothers with low depressive symptomatology, even after adjusting for potential confounders [15, 17]. It is therefore suggestive that the identification and the treatment of maternal depressive symptomatology needs to be extended throughout the offspring’s life course and not be restricted to the immediate postpartum period [18, 19].

Maternal depression is reported as an early risk factor for aggressive behavior [9], depressive symptoms [9], smoking tobacco [4] and alcohol consumption in adolescence [7]. Although maternal depression after childbirth and its association with individual risk-taking behaviors in adolescence has been studied previously, trajectories of maternal depression and risk behaviors in adolescence has been poorly explored. There exist multiple complex pathways through which maternal depression is associated with risk behavior in offspring, including genetic, neurobiological and social pathways. The aim of this study is to evaluate whether the course and severity of maternal depressive symptomatology impacts on risk behavior at age 11, considering potential confounders. We hypothesized that adolescents exposed to high levels of maternal depressive symptomatology throughout childhood and adolescence would engage in more health risk behavior than those exposed to low levels of maternal depressive symptoms.

## Method

### Participants

The 2004 Pelotas Birth Cohort is a population-based birth cohort of children born in the city of Pelotas, Southern of Brazil, from Jan 1, 2004 to Dec 31, 2004. All hospital births throughout that year were identified during daily visits to the city’s five maternity hospitals [20]. 4231 live births of mothers living in the urban area of Pelotas (non-response rate at recruitment was < 1%) were recruited. Trained interviewers administered a structured questionnaire to obtain information on demographic, socioeconomic, biological and behavioral characteristics. The detailed methodology was described elsewhere [20, 21].

### Main exposure: maternal depressive symptoms

Maternal depressive symptoms were assessed by the Edinburgh Postnatal Depression Scale (EPDS) [22]. The EPDS is a self - report, 10-item scale (score range: 0–30, with higher scores indicating more severe depressive symptoms) that expresses the intensity of depressive symptoms over the preceding 7 days. We used a previously translated and validated version of the questionnaire [23]. Follow-up assessments were made at home at mean (standard deviation) ages 3.0 (0.1), 11.9 (0.2), 23.9 (0.4) and 49.5 (1.7) months and at a research clinic at 6.8 (0.3) and 11.0 (0.3) years, with follow-up rates between 87 and 96%. The EPDS was administered to almost all mothers at each follow-up, except at the 3-month follow-up when it was completed by a subsample of 965 mothers. The EPDS scores from 3 months to 11-year follow-up was used to construct the trajectories of maternal depression through a semiparametric group-based modelling approach [24]. We identified five trajectories of maternal depressive symptoms: “low” trajectory group is represented by a linear trajectory comprising women with EPDS< 10 across all time points. The “moderate low” group is represented by a quadratic trajectory comprising mother with EPDS< 10 across all time points. The “increasing” group is composed by women who showed a consistent increase in depressive symptoms during the study period and the “decreasing” group comprises women who showed high EPDS scores in the first 2 years postpartum and a marked decrease afterwards. Finally, the “chronic high” trajectory demonstrated high EPDS scores all through the study period [11, 13, 15, 16].

More details of the steps and methods used to identify the trajectories of maternal depressive symptoms were reported in previous studies [15, 25]. Briefly, 90% of the sample population completed the EPDS at least three times and 17% of mothers completed the EPDS in all follow ups. We included 3347 mothers with data from at least three follow-ups in the analyses.

### Main outcomes: risk behaviors in adolescence

We evaluated two different outcomes of adolescents at age 11. Involvement in fights was accessed via self-report questionnaire with the following question: “Did you get into a fight where someone got injured in the last year?” The answer was analyzed as a binary outcome – yes/ no. Alcohol use was accessed via self-report questionnaire with the following questions: “Have you ever drank alcohol?”. The answer was analyzed as a binary outcome – yes/ no.

### Covariates

Information about maternal, pregnancy, family and child characteristics were collected at the perinatal interview. Socioeconomic and maternal characteristics were: monthly family income in the month prior to delivery (quintiles); maternal schooling (number of completed years of formal education, categorized as 0–4, 5–8, and ≥9 years); maternal self-reported skin color (white or non-white); maternal age at childbirth (< 20, 20–34, ≥35 years old); marital status (single mother or living with a partner); maternal emotional support received from partner during pregnancy (yes/no); parity (the number of previous viable pregnancies resulting in a live birth or a late fetal death, categorized as 0, 1 or ≥2). Smoking and alcohol use during pregnancy were self-reported and were evaluated retrospectively at birth. Women were categorized as having smoked during pregnancy if they reported smoking at least one cigarette per day during any trimester. Women were categorized as having used alcohol during pregnancy if they reported any alcohol use during any trimester. Women were asked when their prenatal care began (first, second or third trimester) and if they planned their pregnancy (yes/no). Maternal mood symptoms during pregnancy was defined as “present” if the mother answered positively to the following question: “During pregnancy, did you feel depressed or have any nervous condition?”. Type of delivery was classified as vaginal or caesarean section.

The father’s presence during child’s life was reported by mothers at 24-month and 48-month follow-up and categorized as “never absent”, “absent sometimes” and “always absent”. Child variables were: sex; low birthweight (< 2.500 g); gestational age (≤36, 37–41 or ≥42 weeks); 5 min Apgar score (< 7 or ≥7); breastfeeding duration was reported by mothers at the 24-month follow-up (≥ 4 month or < 4 months); number of siblings older or younger than the cohort participant assessed at the 11-year follow-up (0, 1, 2, 3 or ≥ 4); child’s hospitalization during 1st year of life (yes/no); Intelligence Quotient (IQ) assessed by the Wechsler Intelligence Scale for Children- III (WISC-III) at age six and categorized as high average (> 0.75 SD), average (− 0.75 to 0.75 SD) and low average (< − 0.75 SD) [26].

### Statistical analysis

We conducted a descriptive analysis of adolescents and mother’s characteristics among those included and not included in the present analyses. Bivariate analysis was conducted to compare characteristics between the two groups. We used chi square test for categorical variables and ANOVA for continuous variables.

All outcomes were analyzed as binary variables. Bivariate analyses were conducted to identify potential confounding variables. Multivariable logistic regression was conducted to evaluate the association between trajectories of maternal depression and risk behaviors at age 11, adjusting for selected confounding variables in separate models. We considered the ‘trajectories of maternal depressive symptoms’ as a proximal determinant of risk behaviors and depression in adolescence with effects that could be confounded by distal variables. Potential confounding variables were included as covariates if they were significantly associated with the main exposure (maternal depression trajectories) and outcome of interest and were not part of the causal chain. They were grouped and included in the adjusted analysis using a backward strategy selection [27]. Five models were included for each outcome: unadjusted results (model 1), model 1 + socioeconomic variables (model 2), model 2 + maternal variables (model 3), model 3 + pregnancy and delivery variables (model 4) and model 4 + paternal and child variables (model 5). If the significance level was below 0.20, the variable remained in the model as a potential confounder for the next level. All analyses were performed with Stata software version 14.2 (StataCorp LP, College Station, Tex).

The study was approved by the Research Committee of the University of São Paulo School of Medicine, and by the Research Ethics Committee of the Federal University of Pelotas. Written informed consent was obtained from the mothers or legal guardians of the adolescents. At the 11-year follow-up, adolescents signed an informed consent form.

## Results

### Attrition analysis

The original cohort has 4231 participants, and of those, 98 died in the first 11 years of life and 3566 were interviewed at 11 years. Data about the outcomes and main exposure were available for 3347 individuals, representing 79,1% of the original cohort. Women who were included in the present study were highly educated; less likely to be single, to be in the lowest quintile of income, and to present mood symptoms during pregnancy. The proportion of women that started prenatal care in the first semester of pregnancy was higher among those included than those not-included. Included and nonincluded women did not differ significantly in age, alcohol and tobacco consume during pregnancy. We also noticed a similar proportion of those mothers who reported that they had planned their pregnancies. Adolescents included in the present analyses had higher birthweight and lower frequencies of 5 min Apgar score < 7 and prematurity than those not included **(**Table 1**).**

**Table 1:** Comparison of maternal and child characteristics between those included and not included in the present study, 2004 Pelotas Birth Cohort

Variables	Included (***n***=3347)	Not included (***n***=882)	***p***-value*
Family income, lowest quintile(%)	19.3	25.5	**< 0.001**
Schooling (years), mean (sd)	8.1 (3.4)	7.8 (3.7)	0.002**
Maternal skin colour, white(%)	73.3	72.0	0.930
Maternal age (years),mean (sd)	26.2 (6.8)	25.5 (6.5)	0.118**
Single mother(%)	15.4	19.9	**0.001**
Parity≥ 2(%)	33.5	37.2	0.079
Smoking during pregnancy(%)	74.9	72.5	0.156
Alcohol during pregnancy(%)	3.1	4.1	0.150
Started prenatal care in the 1sttrimester (%)	73.9	68.8	**0.011**
Planned pregnancy(%)	43.8	41.4	0.205
Mood symptoms during pregnancy(%)	24.0	28.9	**0.003**
C-section(%)	44.9	47.0	0.275
Child sex, male (%)	51.4	53.5	0.282
Low birth weight(%)	8.3	16.5	**< 0.001**
Preterm birth < 37w(%)	13.4	19.2	**< 0.001**
5 min Apgar score< 7(%)	1.3	5.2	**< 0.001**

*Chi-square test

** ANOVA

Table 5 (supplementary material) shows the differences in socioeconomic, maternal and child characteristics between those participants interviewed in both fifth and sixth follow-up waves (at age 6 and age 11) and those interviewed in the fifth follow-up wave (at age 6), but absent in the sixth follow-up wave (at age 11). Adolescents who attended the fifth follow-up, but did not participate in the last follow-up were more likely to be born from a white woman, belong to families from the lowest quintile of income and to be female.

### Risk behaviors in adolescents (outcomes) - descriptive analysis

Of the 3347 individuals included in the present study, 475 (14.1%) have been involved in fights and 269 (8.0%) have already used alcohol **(**Table 2**).**

**Table 2:** Adolescent’s outcomes at age 11 by trajectories of maternal depression, *N*=3347

	Low *N*=1096 (N,%,CI95%)	Moderate Low *N*=1417 (N,%, CI95%)	Increasing *N*=375 (N,%,CI95%)	Decreasing *N*=295 (N,%,CI95%)	Chronic High *N*=164 (N,%,CI95%)	Absolute frequency *N*=3347 (N,%,CI95%)	Frequency(%) (%,CI 95%)
**Involvement in fights**	140 (12.6, 10.8–14.7)	208 (14.5, 12.8–16.4)	61 (16.1, 12,7–20.2)	48 (15.6, 11.9–20.1)	19 (11.5, 7.4–17.4)	475 (14.0)	14.0 (12.9–15.2)
**Alcohol use**	78 (6.9, 5.6–8.6)	122 (8.5, 7.1–10.0)	39 (10.2, 7.6–13,7)	24 (7.9, 5.3–11,5)	14 (8.3, 5.0–13.6)	269 (8.0)	8.0 (7.1–9.0)

### Maternal depression trajectories

Groups “low” representing 32.7% (CI 95% 31.2–34.3) of the women and “moderate-low” containing 42.3% (CI 95% 40.7–44.0) of the women, included mothers with EPDS scores below 10 across all time points. Group “increasing” included 11.2% (CI 95% 10.2–12.3) of the women that had a consistent increase in depressive symptoms during the study period. The group “decreasing”, containing 8.8% (CI 95% 7.9–9.8%) of the sample, was constituted by women that showed high EPDS scores in the first 2 years postpartum and a marked decrease afterward. The group “chronic high” included 4.9% (CI 95% 4.2–5.7%) of the sample and represents those mothers with high EPDS scores across the 11-year period.

### Other factors associated with maternal depression trajectory membership

We investigated whether maternal depression trajectory groups differed on maternal and child characteristics (Table 3). Women in the “chronic high” depression trajectory group were more frequently multiparous, more likely to have an unplanned pregnancy and more likely to report low emotional support received from their partners during pregnancy. They also reported the highest frequencies of mood symptoms and alcohol and tobacco consumption during pregnancy.

**Table 3:** Maternal and child characteristics according to maternal depression trajectory

Variables	Maternal depression trajectory 3 m-11y (N, %)					***p***-value*
	“Low” ***N***=1096	“Moderate Low” ***N***=1417	“Increasing” ***N***=375	“Decreasing” ***N***=295	“Chronic high” ***N***=164	
**Maternal variables**						
Family income (5^th^quintile)	26.9	18.6	11.2	10.8	8.5	**< 0.001**
Schooling>=9 years	55.6	45.4	25.4	28.3	17.7	**< 0.001**
Skin colour, White	76.9	73.3	64.8	69.4	69.5	**< 0.001**
Single mother	12.5	16.5	17.8	18.3	14.6	**0.015**
Maternal age < 20 years	15.2	20.1	22.6	24.7	14.6	**0.001**
Parity>=2	27.9	31.1	42.1	37.9	64.6	**< 0.001**
No emotional support during pregnancy	10.8	16.4	22.0	23.2	26.5	**< 0.001**
Unplanned pregnancy	50.7	55.8	62.1	63.0	68.2	**< 0.001**
Smoking during pregnancy	17.6	24.4	34.1	35.2	42.0	**< 0.001**
Alcohol use during pregnancy	2.0	3.0	2.9	4.0	9.1	**< 0.001**
Depression during pregnancy	10.0	23.4	36.5	42.0	61.5	**< 0.001**
Started prenatal care in the 1sttrimester	79.3	72.7	70.7	68.0	65.4	**< 0.001**
C-section	47.8	44.2	41.6	44.0	42.0	0.182
**Child variables**						
Sex, male (%)	50.5	52.7	53.0	50.1	45.7	0.406
Low birthweight	9.3	7.0	9.6	9.8	7.3	0.181
Preterm birth (< 37 weeks)	11.6	12.9	17.6	15.2	17.0	**0.021**
5 min Apgar< 7	1.7	0.9	2.1	0.6	1.8	0.239
Sibling number>=4	3.4	4.7	6.7	5.9	10.0	**< 0.001**
IQ low average	16.7	23.8	29.5	31.5	37.8	**< 0.001**
Hospitalization 1st year of life	13.7	17.8	21.0	24.7	23.9	**< 0.001**
Breastfed < 4 months	22.2	21.9	26.0	24.6	32.7	**0.018**

*X^*2*^Test

Regarding child characteristics, low birthweight, sex and Apgar score did not vary by maternal depression trajectories groups. Having four or more siblings, presenting a low average IQ and a breastfeeding duration less than 4 months were more frequent among those adolescents whose mother presented a “chronic high” depression trajectory.

### Risk behaviors at 11 years and maternal depression trajectories

We conducted logistic regression analyses to examine the crude and adjusted association between maternal depressive trajectory groups and adolescent risk behaviors (Table 4). We found that ‘involvement in fights’ and ‘alcohol experimentation’ were not associated with any specific trajectory of maternal depression.

**Table 4:** Crude and adjusted logistic regression analysis for risk behaviors (involvement in fights and alcohol use) according to the trajectories of maternal depressive symptoms (children from mothers in “low” group = reference), 2004 Pelotas Birth Cohort

	Models	Low OR(95%CI)	Moderate low OR(95%CI)	Increasing OR(95%CI)	Decreasing OR(95%CI)	Chronic high OR(95%CI)	***P***-value
**Involvement in fights**	Model 1	1.0	1.2 (0.9–1.5)	1.3 (1.0–1.8)	1.3 (0.9–1.9)	0.9 (0.5–1.5)	0.308
	Model 2^a^	1.0	1.1 (0.9–1.5)	1.3 (1.0–1.9)	1.3 (0.9–1.9)	0.9 (0.5–1.5)	0.293
	Model 3^b^	1.0	1.1 (0.9–1.4)	1.3 (0.9–1.9)	1.3 (0.9–1.8)	0.9 (0.5–1.5)	0.432
	Model 4^c^	1.0	1.1 (0.9–1.4)	1.2 (0.9–1.7)	1.2 (0.8–1.7)	0.8 (0.4–1.3)	0.845
	Model 5^d^	1.0	1.1 (0.8–1.4)	1.3 (0.9–1.9)	1.1 (0.7–1.7)	0.8 (0.4–1.4)	0.766
**Alcohol use**	Model 1	1.0	1.2 (0.9–1.7)	1.6 (1.0–2.4)	1.1 (0.7–1.9)	1.1 (0.6–2.0)	0.329
	Model 2^a^	1.0	1.2 (0.9–1.7)	1.5 (1.0–2.3)	1.1 (0.7–1.8)	1.0 (0.5–1.9)	0.486
	Model 3^e^	1.0	1.2 (0.9–1.7)	1.5 (1.0–2.3)	1.1 (0.7–1.8)	1.0 (0.5–1.9)	0.433
	Model 4^f^	1.0	1.2 (0.9–1.7)	1.5 (1.0–2.3)	1.1 (0.7–1.8)	1.0 (0.5–1.9)	0.525
	Model 5^g^	1.0	1.3 (0.9–1.7)	1.5 (1.0–2.3)	1.1 (0.7–1.8)	0.9 (0.5–1.8)	0.584

**Model 1**=crude

**Model 2**=model 1+socioeconomic variables

**Model 3**=model 2+maternal variables

**Model 4**=model 3+pregnancy and delivery variables

**Model 5**=model 4+paternal and child variables

a.Family income, maternal schooling

b.Mother living with a partner, maternal support during pregnancy

c.Smoking during pregnancy, alcohol use during pregnancy, type of delivery

d.Father’s presence during child life, child’s IQ, hospitalization during the 1^st^ year of life

e. Maternal support during pregnancy

f. Smoking during pregnancy, alcohol use during pregnancy

g. Child’s hospitalization during 1^st^ year of life

## Discussion

Our study observed that alcohol use and/or abuse, as well as involvement in fights during adolescence, were not significantly associated with any specific trajectory of maternal depression neither in the crude nor in the adjusted analyses. Although the consequences of chronic and recurrent maternal depression on offspring risk behavior are well known [28–30], most studies have addressed incompletely the impact of the ‘course’ and ‘severity’ of maternal depression on adolescent. We identified few longitudinal studies that investigated the impact of the trajectories of maternal depression on adolescent’s risk behavior [31–33].

It has been already described in longitudinal studies that maternal depression during the first years of a child’s life is a risk factor for several risk behaviors in adolescence such as alcohol and/or tobacco experimentation; and aggressive behavior [9, 34, 35]. However, to the best of our knowledge, only three studies have investigated the role of longitudinal patterns of maternal depressive symptomatology on adolescent’s risk behaviors [31–33], and only one of them addressed the engagement in risk behavior in early adolescence – at age 11 [35] .

Wickham et al. (2015) [31] demonstrated that adolescents exposed to severe maternal symptoms throughout childhood (4 to 8 years) were more likely to engage in violent and nonviolent delinquent behavior at age 16. This group of adolescents also reported earlier age of onset of cigarette, alcohol, marijuana and hallucinogen use when compared to adolescents of mothers in other depression trajectories groups. Nevertheless, when the exposition to severe maternal depressive symptoms occurs in early adolescence, they did not find an increased risk of engagement in risk behaviors. They demonstrated that ‘severity’ of maternal depressive symptomatology is an important risk factor for risk behavior in adolescents only if it occurs during middle childhood - a sensitive period when the effects of adverse experiences are particularly strong in child’s development. In our study, we did not disentangle ‘timing’ from ‘chronicity’ and further longitudinal studies will be necessary to elucidate the association about the ‘timing’ of maternal depressive symptomatology occurrence and risk behavior outcomes in offspring.

Campbell et al. (2009) [33] investigated the association between trajectories of maternal depression and several outcomes in adolescents including ‘engagement in risky behavior’. The outcome ‘engagement in risky behaviors’ was accessed by a self-reported combining items questionnaire - a measure created specifically for their study. Adolescents were asked how often in the past year they had engaged in a series of mildly to seriously risky behaviors (e.g skipped school without permission, taken part in gang fight, smoked marijuana, sold drugs, had sexual intercourse). They found that, at age 15, adolescents whose mothers were in the chronic, elevated, and stable subclinical latent classes reported engaging in more risk behavior than did adolescents of never depressed mother. However, the authors addressed that they could not explain the mechanism by which severity and chronicity of maternal depression enhance the odds of engagement in risky behavior, and added that, probably, there are many other covariates that could influence the finding but were not considered in their study.

Finally, Flouri et al. (2017) [32] investigated the role of different trajectories of maternal depressive symptoms at child ages 3–11 years in offspring risky behaviors such as delinquency and alcohol use, at age 11, using empirical sex stratified models. The study demonstrated that in females, exposure to chronically high maternal depressive symptoms was associated with the view that alcohol use is harmless. In males, exposure to chronically high maternal depressive symptoms was related to aggressive behavior and alcohol use. They concluded that preventing or treating high maternal depressive symptoms in childhood may be an effective strategy for reducing a range of common risky behaviors in early adolescent males and it may also be useful in reducing alcohol use in early adolescent females. Nevertheless, the authors pointed out that the reason for the finding could be biased. First, because the measure of maternal depressive symptomatology used in the study was a measure of psychological distress rather than depression; second because maternal depressive symptoms also co-occur with family stress and less engaged and supportive parenting that could not be measured but probably influence adolescent’s risky behavior.

Differently from the existing literature [31–33], we observed that the occurrence of ‘alcohol use’ as well as ‘involvement in fights’ did not vary according to any specific trajectory of maternal depressive symptomatology. This divergency may have occurred for several reasons: First, because our analyses were adjusted for several covariates such as paternal, maternal, child, pregnancy and delivery characteristics; and not only for gender and socioeconomic covariates as in other previous studies [31], which attributes more strength to our findings. Second, differently from previous studies [32, 33], we used a validated instrument for accessing the main exposure- maternal depressive symptomatology- and we identified each outcome (risk behavior) individually, not in clusters [33] providing more specificity to our results and justifying, in part, our negative findings. Finally, after attrition analysis, more privileged population was included in our study, and the magnitude of the association between the trajectories of maternal depression and offpring alcohol use and involvement in fights was more conservative.

Our study had some strengths: (1) a large population-based sample with high response rate; (2) longitudinal assessments of maternal depression with a long follow-up time; (3) the use of validated instruments for assessing maternal depressive symptomatology (4) the adjustment for various maternal, familial, and child characteristics that potentially act as confounding variables for the associations being investigated; and (5) the study’s contribution to the scarce literature in which the association between the trajectories of maternal depressive symptoms and engagement in health risk behaviors at age 11 are discussed. The study also has some limitations. As nearly 20% of original cohort were not included in the analysis due to missing data, selection bias may have been introduced in our study. In the attrition analysis, we noticed that families with less purchasing power were excluded. We believe that as more privileged were included in our study, the impact of maternal depression trajectories in offspring behavior was probably underestimated and the magnitude of association found in our study is probably more conservative. Another limitation is the choice of a group-based model instead of a longitudinal growth model approach in the construction of the main exposure, which may have underestimated the importance of the predictive factors of the trajectories of maternal depression. Finally, there exist additional unmeasured risk factors that can act as potential confounders of the examined association and were not considered because data on these factors was not collected (ie. familial history of psychopathology and other diseases). This fact contributes to ‘residual confounding’ - an inherent issue of observational studies, but also a limitation of our study.

## Conclusion

Differently for the scarce existing literature regarding trajectories of maternal depressive symptomatology and risky behavior in adolescents, we found no significant association between any trajectory of maternal depressive symptomatology and ‘involvement in fights’ and ‘alcohol use’ at age 11. Further associated factors should be investigated with the aim of preventing alcohol use and/or involvement in fights in early adolescence.

## Supplementary Information

**Additional file 1: Table 5.** Comparison of maternal and child characteristics between those interviewed in both fifth and sixth follow-up waves (at 6 and 11 years old) and those interviewed in the fifth follow-up wave (at 6 years old) but absent in the sixth follow-up wave (at 11 years old) in the present study, 2004 Pelotas Birth Cohort.

## Data Availability

This article is based on data from the 2004 Pelotas Birth Cohort study, conducted by the Postgraduate Program in Epidemiology of the Federal University of Pelotas, Brazil, but restrictions apply to the availability of these data, which were used under license for the current study, and so are not publicly available.
