# Supplementary material for: Trajectories of maternal depressive symptoms and offspring’s risk behavior in early adolescence: data from the 2004 Pelotas birth cohort study
Source: BMC Psychiatry. 2021 Jan 7;21:18. doi: 10.1186/s12888-020-03026-9 (PMC7792177; doi:10.1186/s12888-020-03026-9)
Supplement: Supplementary file 1 — Additional file 1: Table 5. Comparison of maternal and child characteristics between those interviewed in both fifth and sixth follow-up waves (at 6 and 11 years old) and those interviewed in the fifth follow-up wave (at 6 years old) but absent in the sixth follow-up wave (at 11 years old) in the present study, 2004 Pelotas Birth Cohort. [file 12888_2020_3026_MOESM1_ESM.docx]

| **Variables** | **Interviewed in both fifth and sixth follow-up waves**  **(n = 3417)** | **Interviewed in the fifth follow-up wave but absent in the sixth follow-up wave**  **(n = 252)** | **p-value*** |
| --- | --- | --- | --- |
| Family income, lowest quintile (%) | 19.2 | 26.9 | 0.001 |
| Schooling (years), mean (sd) | 8.1 (3.4) | 8.1 (3.9) | 0.897** |
| Maternal skin colour, white (%) | 73.0 | 79.0 | 0.038 |
| Maternal age (years), mean (sd) | 26.2 (6.9) | 24.8 (6.2) | 0.015** |
| Single mother (%) | 15.5 | 17.8 | 0.329 |
| Parity ≥2 (%) | 33.4 | 35.3 | 0.082 |
| Smoking during pregnancy (%) | 24.7 | 29.4 | 0.103 |
| Alcohol during pregnancy (%) | 3.1 | 4.0 | 0.483 |
| Started prenatal care in the 1st trimester (%) | 74.1 | 70.3 | 0.414 |
| Planned pregnancy (%) | 43.8 | 48.0 | 0.190 |
| Mood symptoms during  pregnancy (%) | 24.2 | 30.5 | 0.067 |
| C-section (%) | 45.6 | 46.8 | 0.705 |
| Child sex, male (%) | 59.9 | 51.3 | 0.008 |
| Low birthweight (%) | 8.9 | 8.7 | 0.903 |
| Preterm birth (<37 w) (%) | 13.9 | 11.9 | 0.578 |
| 5-minute Apgar score, <7 (%) | 1.5 | 2.0 | 0.625 |

**Table 5 (supplementary) - Comparison of maternal and child characteristics between those interviewed in both fifth and sixth follow-up waves (at 6 and 11 years old) and those interviewed in the fifth follow-up wave (at 6 years old) but absent in the sixth follow-up wave (at 11 years old) in the present study, 2004 Pelotas Birth Cohort**

*chi-squared test; ** ANOVA test
